# University Clinique

**Published:** 1854-01

**Authors:** S. D. Gross

**Affiliations:** Professor of Surgery in the University of Louisville


### Art. III.—UNIVERSITY CLINIQUE.—SURGICAL CASES AND OBSERVATIONS.

BY S. D. GROSS, M. D., PROFESSOR OF SURGERY IN THE UNIVERSITY OF LOUISVILLE.

It will be recollected by the readers of this Journal that, two years ago, a Surgical Clinique was established by the Medical Faculty of the University of Louisville, and placed under my charge and direction. The object was to furnish, gratuitously, advice, medicine, and operations to the poor of the city, and to such persons from a distance as might resort hither for professional aid without being in a condition to afford adequate remuneration for such services as they might require. The effort, during the first winter, was eminently successful, and would, probably, have been equally so last winter, had the clinique not been suspended on account of my being obliged to take charge of the surgical ward of the Louisville Marine Hospital, which, every alternate session of the school, is under the supervision of the Medical Faculty of the University. A large number of cases, many of them of a highly interesting and instructive character, were presented to the medical class, accompanied by such remarks as their peculiar features seemed to demand, or the occasions prompted. Of the various operations that were performed it is necessary here only to enumerate the more important. These were, lithotomy—three cases—amputation of the leg below the knee; tenotomy; operations for cataract, strabismus and anchylosis; staphyloraphy; the removal of sequestra; and the extirpation of different kinds of tumors from the scalp, neck, and other regions of the body. Almost every form of syphilitic and ophthalmic diseases was exhibited, and commented upon during the course of the session.

The present clinique was opened early in October, immediately after the commencement of the Fall course of lectures, and the pupils of the University have already had an opportunity of witnessing a large number of valuable and instructive cases, as well as a number of important operations. It will be my design, in the ensuing pages, to give an account of some of these cases, with such remarks as they were calculated to elicit at the time of their exhibition; hoping thus to render them more interesting and valuable to the readers of the Journal. It is proper to add, in this connection, that the Clinique is held every Saturday from eleven to one o'clock at the amphitheatre of the University, and that it is open to all the matriculated pupils of the institution, as well as to the regular practitioners of the city, to several of whom, especially to Dr. D. D. Thomson, Dr. D. O'Reilley, Dr. Lyle, and Dr. Metcalf, the establishment is indebted for important contributions in the way of clinical material. My assistants, during the present session, are Dr. Charles S. Savage, of Germantown, and Dr. William R. Evans, of McAffee, Kentucky. The former is the clinical clerk, and upon him is devolved the duty of keeping a record of every case brought before the class. Of the memoranda thus supplied, I shall freely avail myself in the ensuing pages. The reports will be continued in future numbers of the journal.

---

Case 1.—*Hydrocele of the Vaginal Tunic, with Chronic Enlargement of the Testicle; Operation with the Seton; Cure.*

J. McK., of Indiana, aged thirty, was presented at the Clinique on the 3d of October, on account of an enlargement on the right side of the scrotum, caused by chronic swelling of the testicle, and by effusion of serum into the vaginal tunic. The enlargement of the gland had existed for many years, without any tendency to increase, until several months ago, when, in consequence of a blow upon the part, it became quite tender, inflamed and tumid. The injury was so severe as to produce syncope. From this time on, the tumor gradually augmented in volume, and, when the patient reached town, it was found to be at least six inches in length, extending as high up as the external abdominal ring. It was nearly cylindrical in shape, but somewhat larger below than above, free from pain and discoloration, and remarkably firm in its consistence. Its base was formed by the testicle, which was, perhaps, three times the normal bulk, and of uniform outline. The spermatic cord was easily distinguishable at the superior extremity of the tumor, and appeared to be perfectly natural in shape and consistence. There was no enlargement of the subcutaneous veins, or of the lymphatic ganglions of the groin. The opposite testicle was sound, and the general health good.

The absence of fluctuation and translucency of the tumor, together with its extraordinary firmness, threw some little temporary doubt upon the diagnosis; but this was soon dispelled by the introduction of an exploring needle, the puncture of which was immediately followed by the escape of serum, thus revealing the true character of the affection. The instrument having been inserted at several points, the greater portion of the fluid flowed out into the cellular tissue of the scrotum, where it was gradually absorbed, followed by great diminution of the swelling. By the end of a week, however, the accumulation was nearly as great as ever, notwithstanding the steady maintenance of the recumbent posture, the careful suspension of the part, the application of refrigerant lotions, and the internal use of the antimonial and saline mixture. Finding this tendency to recurrence of the disease, the man was taken before the class, and a seton introduced into the tumor with a view to a radical cure. The patient was placed upon his back, when the tumor was seized with the left hand, applied to its posterior surface, so as to render the integuments perfectly tense in front. A medium-sized trocar was then inserted just above the enlarged gland, and after the fluid was evacuated the instrument was re-introduced, and brought out about an inch and a half above the former opening. The trocar was now withdrawn a second time, while a silver probe, armed with a narrow strip of muslin, was passed through the canula protruding at the two orifices.

The tube being removed, the ends of the seton were tied, and the patient, with the testicle properly suspended, was sent to his lodgings. The operation was performed on the 10th of October, and the foreign substance was withdrawn on the third day after, a sufficiency of inflammation having been induced to obliterate the cavity of the sac. During the treatment the man was confined to the recumbent posture, and kept upon light diet. On the fourth day he took an ounce of Epsom salts, and this was all the medicine he used. Cold water was freely employed upon the part, to moderate inflammatory action.

On the 18th of October the patient was discharged cured. The testicle was considerably diminished in volume, and to insure its further decrease I directed the man to pay special attention to his diet and bowels, to keep at rest, and to take, three times daily, a pill composed of three quarters of a grain of iodide of mercury, one twelfth of a grain of tartrate of antimony and potassa, and a third of a grain of opium; continuing its use until the gums should become affected.

In calling the attention of the class to this mode of treatment, I may say a few words respecting the manner in which it effects a cure. The inflammation resulting from the contact of the foreign body eventuates in an effusion of plastic lymph—the animal glue of the system—by which the sac, formed by the vaginal tunic, and containing the serous fluid which constitutes the disease, is ultimately obliterated. I cannot agree with Mr. Liston, Mr. Miller, and some other writers, that the cure is established by a mere change in the secretory powers of the affected membrane; on the contrary, there is reason to conclude, both from analogy and observation, that it depends upon the actual adhesion of the sides of the sac, just as happens in cases of pleurisy where the effused lymph produces adhesion between the pulmonary and costal surfaces. In time, much of this adhesion disappears, but rarely to an extent sufficient to admit of a re-accumulation of serum.

Of all the operations performed for hydrocele, I prefer the seton, for the reason, first, that it is always free from danger, and secondly, that it is always certainly successful. I have employed it frequently, and have never, except in one instance, known it to fail. In that case it would no doubt have been perfectly successful, had it been retained sufficiently long to excite the requisite degree of inflammation. The period of its sojourn in the parts must necessarily depend upon circumstances, of which the principal are the age and volume of the tumor, the health of the patient and his susceptibility to inflammation, the size of the seton, and the manner of performing the operation. In my own practice I have found the average period to be from three to five days.—Generally speaking, the tape should be withdrawn as soon as the part has acquired a considerable portion of its former bulk and consistence, and the skin is red, tense, and painful, or matter appears at the orifices made by the trocar.

The use of the seton is not a modern device in the treatment of this affection; on the contrary, it was resorted to centuries ago. Lanfranc, a surgeon of Milan, appears to have been the first to employ it. Having made a small incision into the skin, he plunged a needle into the tumor, and thus deposited the foreign substance, by the side of which the accumulated serum gradually drained off. Finally, in accordance with the barbarous notions of the times in which he lived, he applied the hot iron to the wound to induce it to suppurate and cicatrize. Guy de Chauliac had recourse to the same treatment; and the seton, without the cautery, was afterwards employed by Ambrose Paré, Peter Franco, and other surgeons in the sixteenth century. In the last century the great advocate of this procedure was Percival Pott, of England, as may be seen by a reference to his able "Tract upon Hydrocele," in his Chirurgical works, edited by Mr. Earle, of London.

The operation by incision I consider not only as dangerous, especially when the system has not been thoroughly prepared, but as incapable, at least in some cases, of effecting a radical cure. I have seen several instances wherethis operation, performed by other surgeons, has been followed by relapse; the adhesions, consequent upon it, having given rise to the formation of several compartments, or chambers, each of which afterwards required a separate operation for its obliteration. In one case, in which I, myself, was the operator, the patient, a stout, robust man, upwards of sixty years of age, came very near losing his life from the severity of the resulting inflammation.

The operation by injection, so generally pursued at the present day, and so much vaunted by some writers, although much safer than the operation by incision, is by no means free from objection, or devoid of danger. Thus, in performing the operation, the canula may slip out of the vaginal tunic, and allow the fluid to escape into the cellular tissue of the scrotum, where its presence may readily induce gangrene and other mischief. Again, I have known the operation to be followed by violent inflammation and suppuration of the vaginal tunic, and, in one instance, even by fatal tetanus. The injection had been performed with port wine and water, and the patient, whom I saw in consultation, was a stout, robust mechanic, about twenty-six years of age. Tetanus supervened on the eighth day after the operation, and in less than two days the man expired. The vaginal tunic was found considerably thickened, and its cavity contained several ounces of sero-sanguinolent fluid, intermixed with unhealthy looking pus. No adhesion had taken place between the opposite sides of the sac, which was highly inflamed. Several similar examples are mentioned by surgical writers.

I have thus spoken of some of the operations which are employed for the radical cure of this affection. Before I dismiss the subject, I wish to offer a few remarks upon one or two other points, a knowledge of which will not be devoid of interest in a practical sense.

In the first place, the tumor in this case is of peculiar shape. Ordinarily it is somewhat pyriform, or pear-like, larger below than above, but here it is perfectly cylindrical, tapering a little as it approaches the groin. Again, the testicle is generally situated at the posterior part of the tumor, about the junction of the inferior with the two superior thirds, but in the case of McK. it lies at the very base of the swelling, which, indeed, it forms. This, then, is another exception to the rule, and one which, diagnostically speaking, is of some importance. The exception here noticed is generally recognized by surgical writers, but not, I think, to the extent it should be. I have paid particular attention to this subject, and am astisfied that the testicle is not unfrequently, instead of rarely, situated at the bottom of the tumor. What the precise relative proportion is my experience does not inform me, but I am sure that I have met with at least eight or nine cases of it within the last few years.

Another feature of some interest in the case is the absence of translucency, which, ordinarily, forms so conspicuous a trait in a tumor of this kind. This circumstance was probably owing to the thickening of the vaginal tunic, produced by chronic inflammation, and thus rendering a structure, naturally transparent, perfectly opake. Finally, the tumor was more firm than we usually find it; generally it is soft, compressible and elastic, often, indeed, distinctly fluctuating, especially in its earlier stages, and when of small size.

The above circumstances, together with the enlargement of the testicle, were, as was before stated, calculated to obscure the diagnosis, and cause me to entertain, at first, some anxiety about the issue of the case. The introduction of the exploring needle, however, at once dispelled these doubts and apprehensions, and led not only to a full comprehension of the nature of the malady, but to a proper method of treatment.

It is interesting to know that hydrocele occasionally disappears spontaneously, or under the influence of slight external injury. Many cases are reported where the fluid was gradually absorbed without any subsequent reproduction, in consequence of the effects of protracted and exhausting diseases; and, some years ago, my colleague, Prof. YANDELL, met with an instance in an elderly gentleman who was cured of a large hydrocele, that had annoyed him for many years, by the concussion of a fall backwards from his horse.

---

CASE 2.—*Un-united Fracture of the Humerus, successfully treated by external Friction, the Splint, and Bandage.*

An Irishman, thirty years of age, of good constitution, and tolerably regular habits, while engaged, on the 7th of July last, in carrying a bale of cotton on his shoulder, fell with his burden, and broke his arm about four inches above the elbow joint. The fracture was transverse. A surgeon being called in, the limb was dressed, and after an attendance of five weeks it was ascertained that the fragments had partially united, but so irregularly as to produce serious deformity. What still further aggravated the case was anchylosis of the elbow-joint. Another practitioner was was now consulted, who, seeing the deformity of the limb, broke the osseous adhesions, and re-adjusted the extremities of the fragments, supporting the parts with splints and bandages. The dressings were frequently removed, and the ends of the broken pieces rubbed against each other, to promote their re-union, but without success. In this condition the patient presented himself at the University Clinique on the 3d of October, nearly three months after the receipt of the injury. There was at this time considerable provisional callus at the seat of the fracture, but the parts were very movable, and it was evident that there was great danger of the formation of an artificial joint. The general health appeared to be good, and there was no inflammation in the soft parts, which could be handled quite roughly without exciting pain or suffering. The elbow-joint was very stiff.

The treatment consisted in washing the arm frequently with soap and water, in rubbing it with spirits of camphor, or some other stimulating lotion, and in supporting it, in the intervals, with a bandage and two leather splints, adapted to the shape of the limb; the hand and fore-arm being carried in a sling. The bowels were maintained in a soluble condition by the occasional exhibition of mild aperients; a light but nutritious diet, with frequent exercise in the open air, was enjoined; and the elbow was moved every few days to restore its functions. Under this treatment there was a gradual but decided improvement, and the man left me at the end of six weeks, with a pretty firm union.

It should be remarked that there was some reason to believe that the man was laboring under a syphilitic taint of the system; though, in this respect, no very satisfactory information could be elicited. I, however, put him, some weeks after he came under my charge, upon the use of the iodide of potassium, giving him ten grains, three times a day. He was still taking the remedy when he left me, and I have reason to believe, from the rapid improvement which occurred soon after the exhibition was commenced, that it rendered him great service, in modifying and improving the action of the capillary vessels of the affected parts.

Want of reunion in a fracture may depend upon a great variety of causes, some of them resident in the parts, others connected with the system. Thus, it may be occasioned by the interposition of a clot of blood, or of a piece of muscle, tendon, or bone. An instance is mentioned where a fracture was prevented from becoming consolidated by the presence of a musket ball. Too much motion, the long-continued use of cold water, especially in persons of a nervous, irritable temperament, and tight bandaging, may also bring about this result. Some years ago I saw a case at the Louisville Marine Hospital, in which, from the latter cause, the consolidation of a fracture of the thigh-bone was delayed for nearly a twelve-month. The limb had become excessively atrophied from the long-continued and injudicious use of the roller, and it was not until after it had been entirely laid aside, and the man had been permitted to exercise upon crutches in the open air, that nature seemed to consider herself in a fit condition to commence the process of reparation, from which she had been so long detained by this mode of treatment.

But the most common local cause of tardy reunion, in injuries of this description, is the want of accurate apposition between the ends of the fragments. This appears to have been the case in the instance under consideration. The memorandum, made by DR. SAVAGE, states that partial consolidation had taken place by the end of the fifth week, but that the deformity was so great that the surgeon, who now took charge of the case, was induced to break the callus, and re-adjust the fragments. Had this defect in the treatment been avoided, the probability is that the re-union would have taken place nearly in the usual time, even supposing that the man's system was slightly tainted with the syphilitic poison.

The great error, commonly committed by practitioners in the treatment of fractures of the shaft and lower extremity of the humerus, is, that they do not afford sufficient dependency to the elbow. Instead of permitting this part of the limb to hang down they support it in a sling, and consequently force the inferior fragment out of its natural relations, beyond the upper one. The proper plan is to keep the elbow free, but steady, so as to compel it by its own weight to maintain the requisite apposition.

The principal constitutional causes which interfere with the reparative process are, debility, whether from loss of blood, want of nutritive action, or exhausting disease, as long-continued fever; a gouty, rheumatic, or syphilitic state of the system; and loss of nervous influence. Another cause, but one which, I presume, seldom, exerts much influence, is pregnancy. It is barely possible, I conceive, that during this state there may be such an abstraction of blood from the affected parts for the nourishment of the fœtus as to retard, and, perhaps, even temporarily prevent, the formation of callus. In the few cases of this kind which have fallen under my observation, I have not, however, witnessed such an effect, and I am strongly inclined to believe that this influence has been greatly magnified, if indeed, it is not almost wholly chimerical. Another cause, probably much more efficient, as well as much more common, than the one just alluded to, is the protracted and inordinate use of ardent spirits, weakening the nutritive energies of the system, and rendering the blood and its vessels unfit for the performance of the important duties assigned to them in the reparative process.

Whatever the cause may be, great pains should always be taken to discover it, with a view to its early and efficient rectification. Should it consist in debility, however induced, the patient should be at once put upon the use of nutritious food and drinks, as porter, ale, wine, or brandy, aided, if necessary, by tonics, of which iron and quinine are generally the most eligible. A gouty or rheumatic state of the constitution is best remedied by purgatives, acid drinks, and colchicum. Tertiary symptoms should be met by iodide of potassium, either alone, or in union with mercury, the latter of which should sometimes be carried to the extent of slight ptyalism. Debility from drunkenness must be counteracted by the judicious employment of ardent spirits, such, if possible, as the patient has been in the habit of using previous to the accident.

The local treatment must be regulated by the circumstances of each particular case. The precise cause of the tardy or imperfect union must, if possible, be clearly ascertained, and immediately remedied by appropriate measures. If it depends upon too much motion, greater quietude must be insured; any defect of contact must be redressed by a more accurate adjustment of the ends of the fragments; cold applications, if injurious, must be discontinued; and any extraneous intervening substance must be removed, either by calling into requisition the agency of the absorbent vessels, by pressure and other means, or, as in the case of a piece of dead bone, by the knife and forceps. The cause of the defective union having been thus remedied, the case will be likely, of its own accord, to proceed to a favorable termination, the ordinary principles of treatment being, of course, observed.

The principal local remedies, beside those just mentioned, are: 1. Cutaneous friction, either with the bare hand, or with a piece of flannel; and the friction may be either dry or moist. In the latter case, various liniments, lotions, or unguents may be employed, and often with marked benefit, inasmuch as they tend to excite the action of the capillary vessels in and around the ends of the fragments, and thus promote the formation of callus. 2. Compression, steady and persistent, uniform and gentle; performed by means of leather splints and a bandage, as in the case of our patient; or by an apparatus expressly constructed for the purpose, and intended to concentrate the pressure at the seat of the fracture. 3. Blisters and iodine; these remedies are employed in the usual manner, and are mainly useful when the defect is dependent upon undue vascular excitement. 4. Friction of the ends of the fragments against each other, as recommended by CELSUS, and practised by modern surgeons. The operation should be performed very gently, and be repeated once every four, six, or eight days, according to its effects, care being taken to confine the limb in the intervals by an appropriate apparatus. 5. Acupuncturation with a long, slender needle may be tried; or a small incision may be made over the seat of the injury, and a heated wire inserted between the ends of the fragments. These are modern devices, which have occasionally been followed by success. 6. The seton; introduced into practice, in 1802, by the late Dr. PHYSICK, of Philadelphia, and ordinarily, in obstinate cases, the most certain method. It should be passed between the ends of the broken pieces by a long, thin, flat needle, sharp and lancet-shaped at the point; or, where this is impracticable, as near the site of the fracture as possible; for experience has shown that this mode of performing the operation is nearly as successful as the usual procedure. The foreign body is retained for a variable period, longer in some cases than in others, and generally until it has excited suppurative action. The patient is carefully watched; and if the pain and swelling become severe the seton is at once withdrawn. Immediately after the introduction, the fragments are carefully adjusted, and steps taken, if necessary, to main extension and counter-extension. In the first case in which this treatment was employed, the seton was retained many weeks, and the patient recovered the perfect use of his limb. My belief is that the late Mr. Liston was in error when he advised that it should always be withdrawn in a few days, for the reason that it will hardly have sufficient time, in such a case, to excite the requisite degree of inflammatory action. 7. Excision of the ends of the fragments, an operation devised and first performed in 1760, by Mr. White, of England. Such an operation should never be resorted to without due deliberation, and until after the failure of the more ordinary and simple means. To say nothing of the difficulty of its execution, it is by no means devoid of danger; indeed, it has not unfrequently proved fatal. A very free incision is made through the soft parts down to the ends of the broken bone, which are then brought out at the wound, and retrenched, either with a stout knife, a saw, or a pair of pliers. Sometimes the mere removal of the cartilaginous crust is sufficient for the purpose, an object which may be easily accomplished by scraping. Finally, there are some other modes of procedure, chiefly modifications of some of the preceding, which will come up in the regular lectures, and which need not, therefore, be specified on this occasion. I have given you this outline of the local treatment of un-united fracture for the two-fold purpose of showing yon what may be done under such circumstances, and of affording you some idea of the genius and fertility of this particular branch of surgery; for no where do the labors of professional men appear to greater advantage, or in a more striking point of view.

---

### Case 3.—*Traumatic Neuralgia.*

The patient, an Irishman of the name of Madden, aged 60 years, a laborer, of tolerably temperate habits, has had a local pain on the right side of the scalp, just behind the coronal suture, near the top of the head, for thirty years. He recollects that he received a slight hurt on that part of the head some time before the pain made its appearance; but of its precise character he is, at present, unable to give any satisfactory account. The pain is generally periodical, coming on usually about the middle of the day, and continuing, with more or less severity, for six, eight, ten, or even twelve hours, when it gradually disappears, leaving the patient comparatively comfortable until the next attack. Occasionally it is entirely absent for several successive months. Exposure to cold and wet, irregularities of diet, the use of ardent spirits, or derangement of the digestive apparatus, invariably aggravate the local distress, and often cause an attack of it. The pain commonly begins at a small circumscribed spot, hardly as large as the end of the finger, exquisitely tender on pressure, and slightly œdematous. During its progress it extends over a considerable portion of the scalp, and, not unfrequently, even down the neck and shoulders. The general health has long been impaired; the tongue is constantly coated; and the bowels are habitually constipated, requiring the frequent use of medicines. In fact, the man has all the symptoms of a dyspeptic. It is proper to observe that he often complains of neuralgic pains in different parts of his body, that he has occasional attacks of fever, and that he is, now and then, troubled with partial deafness. No chilly feelings precede or accompany the local distress.

The patient having taken some purgative and anti-periodic remedies without any very material benefit, I cut down through the tender and painful spot in the scalp as far as the cranium, and introduced into the incision a small, smooth pebble, for the purpose of inviting a free discharge of matter. After the foreign body had been retained about a week, it accidently fell out of the wound, which, as the distress was measurably relieved, was accordingly permitted to heal. The patient, meanwhile, and for sometime subsequently, was kept under the use of purgatives and antiperiodic remedies; the latter consisting of three grains of quinine, the twentieth of a grain of strychnine, the twelfth of a grain of arsenious acid, and the eighth of a grain of extract of strammonium, with a small quantity of morphia, made into a pill, and repeated every five hours. Under this treatment, the general health greatly improved, and in a few weeks the man went home, nearly entirely free from the local pain which had so long harrassed him.

In commenting upon this case, it may be stated that pain of the scalp, as it presented itself in the person of Madden,—local, circumscribed, and attended with tenderness of the part and derangement of the general health,—is often occasioned by external violence, as a contused, lacerated, or punctured wound, and that, what is remarkable, it sometimes does not appear until months and even years after the occurrence of the injury. A sort of latent irritation is set up, followed, in many instances, by a deposit of lymph, and consequent thickening and induration of the affected tissues. When great and long-continued, this irritation may give rise to various nervous symptoms, as hemicrania, spasmodic twitching of the muscles of the face, partial paralysis of the limbs, and even convulsions. I recollect, among other cases, that of a young gentleman, who, in consequence of an injury of the head, produced by pitching against a brick-bat, used to suffer in the latter way for a long time. When I first saw him he was about seventeen years of age, during the last ten of which he had experienced great local pain and tenderness of the scalp on the right side, near the antero-superior angle of the parietal bone. The patient was slightly stunned by the accident, and the little wound emitted a few drops of blood; but he recovered in a few minutes, and resumed his wonted play as if nothing had happened. At the end of five days, however, he was seized with severe convulsions, which recurred afterwards, at variable intervals, gradually impairing his mind, and rendering him exceedingly irascible and unmanageable. The attacks continued for many years, and were attended by frequent paroxysms of head-ache and fits of the most horrible despondency. The cephalalgia was generally periodical, and was always accompanied by exquisite tenderness of the scalp, often extending over the greater portion of the head, and being particularly severe and distressing on the affected side. The case at length became desperate, and the parents of the young man had serious thoughts of sending him to some lunatic asylum. It was about this time that I was requested to see him in consultation with the family physician. I found, as already intimated, a very tender spot on the upper and fore-part of the side of the head, scarcely as large as a twenty-five cent piece, free from swelling, but so sensitive that the slightest pressure with the point of the finger occasioned the most intense pain and suffering. There was no evidence of depression of the bone or disease of the osseous tissue. The general health was considerably deranged, and the youth was gloomy and morose. Satisfied that the disease depended upon chronic thickening of the pericranium, I at once suggested, as the most probable means of relief, a free division of the scalp at the seat of the local distress. The propriety of this view being concurred in by the medical attendant, the operation was accordingly performed a few days afterwards, the incision being a little over an inch in length, and carried down through the whole thickness of the pericranium. A dossil of lint being inserted, a compress and bandage were applied, and the patient was restricted to light diet. In a few days the wound began to suppurate, and after the discharge had been maintained for some weeks, the parts were permitted to cicatrize. Long before this time, however, the bad symptoms had subsided, the tenderness was entirely relieved, and the patient, afterwards, gradually regained his general health and the use of his mental powers.

Finally, it may be remarked that, while a free division of the affected part is the most prompt and efficacious remedy, other means may occasionally be resorted to with a prospect of temporary, if not permanent, relief. I may instance, in particular, the application of leeches and blisters to the affected part, the establishment of an issue with the Vienna-paste, and the inunction of mercurial ointment, in union with veratria and belladonna. When the disease depends upon the presence of an indurated cicatrice, or thickening of the pericranium, the proper remedy will be excision. In all cases great attention should be paid to the patient's diet, bowels, and secretions. The best internal means, besides purgatives and alterants, are emetics, and quinine and iron, in combination with arsenious acid, strychnine, and extract of stramonium, in the proportions above mentioned.

---

CASE 4.—*Fatty Tumor; Excision; Cure.*

JOE, a negro, aged thirty-nine years, from Boyle county, Ky., was sent to me, early in November last, by Mr. KNOX, a member of the class, on account of a fatty tumor situated in the right groin. From a memorandum made by Dr. EVANS, it appears that the tumor was first noticed, about thirteen years ago, as a small nodule some distance above Poupart's ligament. Its growth was gradual but persistent until about two years ago, when it became much more rapid, and the morbid mass is now upwards of eight inches in length, by more than fifteen in circumference. It is of a flattened conical figure, and hangs off from the thigh, when the body is inclined forward, like a gourd, its attachment being by a comparatively small and rather narrow foot-stalk. To the hand it imparts a doughy, semi elastic, consistent feel. The surface is smooth and uniform, and there is no enlargement of the subcutaneous veins, or unnatural color of the skin. The tumor is free from pain, and causes no other inconvenience save what results from its weight and bulk. There is no disease of the neighboring lymphatic ganglions; and the general health is very good, the man being stout and robust. From a careful examination of the history of the case, I was induced to conclude that the tumor was a fatty one, and accordingly so expressed myself to the medical class.

As the patient was perfectly well, I did not deem it necessary to spend much time in preparatory treatment. Having kept him upon light diet for several days and given him a dose of purgative medicine, I placed him upou the table of the amphitheatre at the University, and by a rapid dissection removed the tumor, taking care to make my incisions several inches above its point of attachment, in order that I might thus obtain a sufficient amount of integument to cover the wound. Scarcely an ounce of blood was lost in the operation. Knowing that but a few minutes would be occupied in the removal of the tumor, I gave the patient no chloroform. The edges of the wound were approximated by three points of the interrupted suture and adhesive strips, supported by a compress and bandage, carried round the hips and thighs. I was kindly assisted in the operation by Dr. T. G. Richardson, the demonstrator of anatomy in the University.

Owing to the peculiar mobility of the parts involved in the operation, only a portion of the wound healed by the first intention. Notwithstanding this, however, the patient was well enough to return home in less than two weeks, having recovered without a single accident.

The tumor, after its removal, was found, as had been previously announced, to be of a fatty nature. It was made up of masses of adipous matter, connected together by cellular tissue, and varying in size from a cherry to a pullet's egg, of a pale yellowish color, and of the ordinary doughy, semi-elastic consistence. In short, its characters could not possibly have been more distinctly defined.

In connection with this case a few remarks may be made respecting the nature and habits of this form of morbid growth.

The fatty tumor is quite common, and should, therefore, be well understood by the practitioner. It may take place in any part of the body, with the exception, perhaps, of the palm of the hand, and the sole of the foot, the fingers and toes. I have seen it, however, more frequently about the back, shoulder, and neck, than in any other region. The upper eye-lid is also a common seat fo it. Sometimes, but more rarely, it occurs in the orbit of the eye, in the walls of the abdomen, in the perinæum, the pudendal lips, and underneath the tongue, and even in the substance of this organ. Large masses of this kind occasionally form in the internal cavities of the body, as in the omentum and mesentery, and around the kidneys. In a specimen which I removed, some years ago, from the body of an old man after death, the growth weighed three pounds, and the late Prof. HORNER, of Philadelphia, refers to one, removed from a bullock, which filled a common sized wash-tub. When the viscera are loaded with masses of fat, they impart to the abdomen that projecting rotundity, known by the name of "pot-belly," so well described by Prince Henry, in his address to Fallstaff, as "a huge hill of flesh," "a globe of sinful continents." In the chest large masses of fat sometimes envelop the pericardium, and, by compressing the heart and great vessels, induce palpitation, and even fatal syncope, as in the interesting case narrated by SENAC.

The number of fatty tumors varies, in different cases, from one to several hundred. In general, they are solitary, or, at most, there are only two or three, occupying different regions of the body, or grouped more or less closely together. In a medical gentleman, aged 23, who attended our lectures some years ago, I counted upwards of two hundred, from the volume of a small pea up to that of a large marble. They all had a doughy, inelastic feel, and nearly all were of a globular shape; a few were slightly flattened, or compressed. They were situated principally on the fore-arms, the inside of the thighs, the loins, abdomen, and pectoral muscles, the latter of which were literally covered with them. None existed on the head, neck, and upper part of the back. The general health was good, and the tumors had been first observed about sixteen years previously. During two severe attacks of acute disease, accompanied with great emaciation, many of them entirely disappeared. To satisfy myself of the true nature of these tumors, the gentleman permitted me to remove one, about the size of a filbert, which proved to be composed entirely of fatty matter.

In their volume, these tumors vary from that of a small pea to that of an adult head. Sometimes, indeed, they are much larger, measuring many inches in diameter, and projecting a great distance beyond the surface. My friend, Dr. Ray, of Evansville, removed one a short time ago which weighed nearly forty pounds, and which, had it been permitted to increase, might have acquired a still greater bulk. Cases are recorded where the weight was nearly sixty pounds. My own practice has never furnished me with such enormous growths.

In their shape, fatty tumors are generally somewhat globular, but as they augment in volume they are liable to become elongated, and to assume a pyriform, gourd-like, or pediculated configuration, as in the case just operated upon. These changes in their form no doubt depend upon their weight, by which they are gradually dragged out of their original shape, as well as position. For the same reason they sometimes shift their seat, descending from the point where they originally appeared to one below it, perhaps several inches distant. Thus, a fatty tumor which was developed in the groin, has been known, in time, to pass down between the scrotum and the thigh. This migratory tendency, which is interesting as a matter of diagnosis, is most common in those parts of the body which are abundantly supplied with loose cellular substance, and in those cases in which the tumor has a large bulk and a pediculated attachment.

Fatty tumors are always, according to my observations, invested with a capsule, by which they are connected with the surrounding structures, and through which they obtain their vessels, nerves, and absorbents. This covering is not a new formation, produced by inflammatory action, but is the result simply of a condensation of the circumjacent cellular substance; and hence it varies very much in its appearance in different cases and in different circumstances. In the early stage of the affection, and especially when the tumor is diminutive, it is, in general, very thin, soft, elastic, and transparent; but in cases of long standing and large size, it is always more or less dense, firm, resisting, and of a fibro-cellular, or distinctly fibrous texture. Its thickness ranges between a mere film and a layer of several lines in depth. External pressure, especially if long continued, and the pressure also of one part of the tumor upon another part, no doubt exert an important influence upon the anatomical character of this investiture, and serve to adapt it to the varying circumstances of the shape and bulk of the morbid growth. The adhesion of the capsule to the skin is sometimes remarkably close, requiring great care in separating it.

Attached to the inner surface of this covering are numerous processes, which dip into the interior of the morbid growth, separating it into lobes, lobules, and granules, until the component tissues are resolved into their ultimate elements. These processes are usually very delicate; but occasionally, as when there is a hypertrophous condition of the fibro-cellular element, they are quite dense and tough, forming distinct bands, of a whitish or greyish color, between the different structures.

Fatty tumors do not receive much blood, at least not as a general rule; and hence they seldom bleed much when they are extirpated. It is only when they are of large size, or when they grow very rapidly, that they are likely to be very vascular. The capsule and its processes serve to conduct the vessels into the interior of the tumor, and to direct, as it were, the distribution of their branches and ramifications. As the morbid mass is always free from pain, and is tolerant of the rudest manipulation, it may be concluded that it receives very few nerves. Its absorbent vessels are also few in number. It may also be inferred that, inasmuch as the general health is usually unimpaired throughout the whole progress of the affection, however long it may continue, it does not possess any important sympathetic relations with the general economy.

It has been already stated that fatty tumors are most common in the external parts of the body, and it may now be added that they are most frequent in the subcutaneous cellular tissue. They are not, however, confined to this substance; for in many cases they send prolongations around the muscles, tendons, fasciæ, vessels, and other structures. Thus a fatty tumor of the neck has been known to extend deeply between the trachea and œsophagus, or to dip in between the carotid artery and jugular vein, or to pass down behind the sternum and clavicle into the chest. A fatty growth of the wall of the abdomen sometimes extends into the cavity of that name; and, on the other hand, such a tumor occasionally begins in the subperitoneal cellular tissue, and ultimately descends through the inguinal canal, or some abnormal outlet, down into the scrotum, thus simulating hernia of the groin. It must be obvious that all such arrangements, which, however, are fortunately rare, must greatly embarrass both our diagnosis and our attempts to remove the morbid mass.

Fatty tumors are soft, doughy, and semi-elastic;—properties which, in general, enable the surgeon readily to distinguish them from other morbid growths. Their boundaries are usually well-defined, especially when they are superficial or pediculated. Sometimes, however, they are insensibly lost in the surrounding parts, being spread out beneath the skin, and sending processes among the muscles, or their fasciculi. In many cases they have an irregular, lobulated surface; while in other, and perhaps in the majority, they are perfectly smooth and uniform. There is no enlargement of the subcutaneous veins, no disease of the skin, no pain, and no tenderness on pressure. The progress of the tumor, in fact, is quite indolent; and the only inconvenience which the patient experiences is what results from its weight and bulk. The general health is usually perfect. These circumstances, with a careful consideration of the history of each case, will commonly serve to distinguish fatty tumors from other morbid growths, whether benign or malignant in their character.

Fatty tumors are liable to inflammation, suppuration, ulceration, and even gangrene. These events, however, are very infrequent, and are usually induced by mechanical pressure, by caustic applications, and by inefficient nourishment, in consequence of a loss of vascular and nervous supply from the pendulous or over-grown character of the morbid mass. In a tumor of this kind, about the volume of an orange, which I removed, many years ago, from the top of the left shoulder of a girl, of eighteen, at Cincinnati, the ulcer had a remarkably foul, unhealthy aspect, with thin, everted edges; the pain was at times quite severe, and the discharge was of a sanious nature, intermixed with globules of fat. Various attempts had been made, but without success, to heal it up, and no cause could be assigned for its formation. The general health had been a good deal impaired, and for some time past there had been irregularity of the menstrual function. The ulcer was included in the incisions, and a speedy recovery was the result.

When matter forms in such a tumor it is rarely that it is collected into a distinct abscess. Of this occurrence, the most remarkable instance of which I have any knowledge is related by Mr. Brodie.

ABERNETHY* saw osseous matter deposited within the substance of a fatty tumor; and a specimen of a similar kind is contained in my private collection. The osseous matter, in this case, consists of a small nodule, not larger than half a marble, very hard, and of a pale yellowish color. AUVERT has described the same change in the sixteenth plate of his splendid Surgical Illustrations. Mr. PAGET refers to a similar specimen in the Museum of St. Bartholemew's Hospital, London.†

Again, such tumors occasionally undergo a sort of fibrous, or cartilaginous degeneration, not uniformly, but at certain points of their extent. Nodules, varying in size from a hazel-nut to a pullet's egg, may thus be formed, having a firm, characteristic consistence, more or less movable, and contrasting singularly with the other structures. Finally, they occasionally contain cysts, filled with various kinds of substances, as oily, serous, or gelatinous. The walls of the cysts may be very thin and transparent, or thick, opake, and perhaps even partially calcified, as in the instance mentioned by PAGET.

It is not easy to determine how fatty tumors are developed.

---

* Surgical Works, vol. 2, p. 300.

† Lectures on Surgical Pathology, vol. 2, p. 100. London—1853.

The difficulty which surrounds the subject is not cleared up by assuming that they are merely hypertrophies of the natural adipous tissue. This is probably the fact; but we cannot explain why such an occurrence should take place at one point rather than at another, or why, indeed, it should happen at all. The exciting cause of the morbid growth has sometimes been traced to external injury, as a blow, contusion, or steady mechanical compression; but in the generality of cases no reason whatever can be assigned for its production.

Both sexes are liable to this formation, but whether in an equal degree or not, is not ascertained. It is most common in young adults and middle-aged persons, being rarely developed in the very young or very old. Animals are also subject to it, and in them it occasionally attains an enormous bulk.

Not much need be said here respecting the medical treatment of this class of morbid growths; for, so far as our knowledge at present extends, there is no article of the materia medica, or combination of articles, that is capable of arresting its progress, or causing its removal. If the reverse is occasionally the case, it is only as an exception to the rule, and nothing else. The instance of Mr. Brodie,* in which that gentleman succeeded in removing a large mass of fat from a man's chin and neck, by the free and persistent use of the solution of potassa, may be regarded as a memorable example of unexpected success. He commenced with half a drachm of the fluid three times a day, and gradually increased the dose to a drachm. At the end of a month there was a sensible diminution in the volume of the tumor, which steadily continued as long as the medicine was persevered in. Sometime afterwards Mr. Brodie substituted the tincture of iodine, which had just then come into use, and the tumor again increased. Finding this to be the case, the alkali was resumed and continued, off and on, until the tumors were almost entirely dispersed. Altogether, the man took an enormous quantity of the medicine. The case of Mr. Brodie is a solitary one; at all events, I am not aware that the same happy effects have been realized from this remedy by other practitioners.

* Select Surgical works, p. 202. Philadelphia—1847.

All local applications are equally unavailing. This is true alike of steady and systematic compression, of mercurial and other inunctions, and of frictions with stimulating liniments and embrocations. The absorbent vessels in these tumors are few and feeble, and this is probably the reason why all remedies of this description are so utterly valueless as curative agents.

From the above remarks we may learn how useless it is to spend our time in idle and unprofitable attempts to cure these deposits by constitutional and topical means. They teach us that the only reliable remedy is the knife, and that the sooner this is resorted to the better it will be in all cases where the tumor is in an ingravescent state, and likely, by its pressure, to interfere with the functions of important organs. As long, on the contrary, as it is small and harmless, causing no inconvenience or disfigurement, it may as well be let alone.

The manner of excising such tumors may be gathered, in some degree at least, from the case already described. Any diseased skin that may exist should be removed along with the morbid mass, which should be enucleated by a rapid dissection, care being taken that not a particle of the deposit be left behind; otherwise a reproduction might readily take place. When the tumor is superficial the operation is easily performed and soon over; but when it is deep-seated, or when it sends processes among the surrounding structures, it may be one of great difficulty and perplexity, requiring the most consumate skill for its successful execution, and the most thorough knowledge of the anatomy of the parts. For the reasons already mentioned, the operation is sometimes nearly bloodless. I have, indeed, seldom found it necessary to apply any ligatures, whatever might be the volume of the tumor.

---

### Case 5.—*Onychia Maligna.*

The present case is one of a deeply interesting nature, both on account of the obscurity of its origin, its real pathology, and its obstinate and intractable character. It consists in ulceration of the root of the nail of the fore-finger of the right hand, and constitutes what Mr. Wardrop has described as onychia maligna. The disease, in the patient before us, was first noticed about four months ago as a small, elongated blister at the sides and back part of the nail, filled with serous fluid, very painful, and surrounded by a reddish inflammatory circle. Upon the rupture of the vesicle, which happened in a few days after, a narrow, superficial, angry looking sore was exposed, followed by an oozing of thin, ichorous fluid, seemingly irritating to the adjacent parts. The ulcer gradually extended in depth and diameter, until it at length involved nearly the whole dorsal surface of the last phalanx, destroying the nail and giving the end of the finger a peculiar bulbous appearance, as if it had been forcibly compressed laterally, at the same time that its superior surface has a hollow, cup-shaped arrangement. A portion of the nail still remains attached at its root, but the connection is very slight, and will probably be entirely destroyed in a few days. The nail is of a dark color, and of a soft, friable consistence. The edges of the ulcer are elevated, thin at some points and thick at others, jagged, and of a purple hue; the surface, as already stated, is depressed, or, as it were, scooped out, and incrusted with yellowish aplastic lymph. Here and there is a small unhealthy granulation, exquisitely sensitive, and evidently unable to make any progress towards reparation. The discharge is occasionally quite abundant, and is generally of a very fœtid character. The bulbous extremity of the finger is very hard, tender, inflamed, and of a dusky, purple complexion.

The child, who is seven years old, is rather thin; and the general health, although much better than it was three weeks ago, when the case first came under my observation, is far from being good. The appetite, however, is much improved, and she rests much better at night. At present, and indeed for the last fortnight, there has been hardly any pain in the part. Prior to that period, the suffering was so intense and constant that the child found it difficult to obtain any sleep at night. The pain was sharp and burning, and generally accompanied with a sense of throbbing. It is worthy of notice that the features are denotive of the strumous diathesis, the complexion being very delicate, and the eyelashes unusually long. The extremities are habitually cold.

Of this affection, which was first described in 1814, by Mr. Wardrop, an eminent English Surgeon, in the fifth volume of the Medico-Chirurgical Transactions of London, I have seen only four well-marked examples in a practice of twenty-five years, and hence I conclude that it is quite rare. The first case occurred in this city in 1843, in a lad, aged eight years, the son of a Mr. Clark, at that time a resident in Grayson street. It involved the great toe of the left foot, and had made such extensive ravages that the affected part had been pronounced as incurable by the professional attendant, by every means, except the knife, to the employment of which, however, the parents had fortunately refused their consent. The disease had been in progress for upwards of eight months; the nail was nearly completely destroyed; the ulcer had a foul, dirty appearance, with thin, everted edges; the toe had the characteristic bulbous shape; and the discharge was thin, sanious, and excessively offensive. The general health was greatly undermined; for the lad was pale and emaciated, had no appetite, and was unable to sleep, owing to the local pain, which sometimes amounted to exquisite torture.

The second case was that of a boy, aged ten years and a half, the son of a Mr. AALEXANDER, late of this city, now of Cincinnati. It occurred last autumn a year ago. The disease, which occupied the right thumb, had made considerable progress before my attention was called to it, and the parts exhibited the peculiar features already more than once alluded to. Excessive pain in the ulcer, the gradual destruction of the nail, an abundant fœtid discharge, and great disorder of the general health, with loss of sleep and appetite, were the leading symptoms in the case. The constitution was naturally feeble, and every thing betokened the existence of a strumous temperament.

The third case was the one described at the beginning of my remarks. For the fourth, I am indebted to Dr. JOHN BARTLETT, of this city, who kindly brought the little patient, an orphan girl, aged four years, before the University Clinique, on the 17th inst. The child, although represented to be in good health, looks pale and delicate, and it is very apparent, from the state of her complexion, eyes, hair, and eye-lashes—which are of unusual length—that she is strumously inclined. Dr. BARTLETT states that about three months ago she received an injury upon the end of the left ring finger, which was followed by inflammation, and, at length, by ulceration of the root of the nail. A large portion of the nail, which is of a dark, dirty, brownish color, still remains, but its attachments are very feeble, and will be entirely destroyed in a few weeks, if not sooner. The ulcer has a foul, excavated appearance, the discharge is thin and ichorous, and the end of the member has the peculiar clubbed or bulbous form. The part is perfectly free from pain, nor has there been any pain in it, at any time, save what was produced by the topical applications employed for the relief of the morbid action. About a week before the child was presented at the Clinique, Dr. BARTLETT thought that the ulcer was in a healing condition; but upon inspecting it again, a few days after, he noticed that it exhibited all its former unpromising features. No constitutional treatment had been resorted to; locally, use had been made successively of nitrate of silver, sulphate of copper, and the nitric acid lotion, without any special benefit.

From the history of the above cases, I am inclined to believe that the disease under consideration is connected with a peculiar scrofulous state of the system, but in what this state consists, or how it is induced, we cannot, of course, determine. Be this as it may, it is always exceedingly obstinate and intractable, frequently very painful and distressing to the patient, and exceedingly perplexing to the surgeon. Its duration varies from a number of weeks to many months. Sometimes the malady continues until it destroys, not only the nail and soft tissues beneath and around it, but bone and everything else. The diagnosis is always easy, particularly after the affection has made some progress. The inflammatory line or circle at the root of the nail, succeeded by the development of one or more vesicles, and the exposure, on the rupture of these vesicles, of a foul, irritable ulcer; the spreading tendency of this ulcer, and finally, its excavated appearance; the discolored and sloughing condition of the nail; the sanious, eroding, and fœtid discharge; and, above all, the singularly bulbous form of the distal extremity of the affected member, whether toe or finger; leave no doubt in regard to the true nature of the case. Once seen, such a state of things can never be forgotten.

Onychia maligna is most frequent in children from the age of five to twelve years. In the cases already described, the ages of the patients were, respectively, four, seven, eight, and ten and a half years. Mr. WARDROP states that, while the affection is most common in early life, he has often seen it in adults. My distinguished friend, the late Professor JOHN B. BECK, of New York, who, many years ago, published an account of five cases of onychia maligna, found the ages of his patients to be, respectively, five, six, ten, ten, and twenty-three years. Two were males and three females. The disease in one of the cases occupied the great toe, in one the thumb, and in the other three the finger. In three of the cases the ulceration was preceded by external injury; in the remainder it commenced without any assignable cause. In all the instances that have come under my own observation, save one, it seems to have had a spontaneous origin. In Dr. Beck's cases, none of the patients complained of much pain; in three of mine, on the contrary, the pain was a constant and prominent symptom. Mr. Wardrop observes that the pain is sometimes very acute, but that the disease is more commonly indolent, and accompanied with little uneasiness. He also remarks that he has seen the ulcer more frequently on the thumb than on any of the fingers and toes.

The term malignant, as applied to this disease, is ill-chosen; because, while it does not express its true character, it is apt to lead to erroneous notions respecting its curability. That the malady may, if permitted to have its own way, eventuate in the total destruction of the affected part, is unquestionable; but such a result is not to be regarded, in any case, as a necessary consequence of the malady, and may, almost invariably, be prevented by the timely interposition of proper remedies. Moreover, when once removed, the disease never returns, as happens in the various forms of malignant affections. The malady is obstinate and rebellious, distressing and annoying to both patient and practitioner, but never malignant. It is important that this point should be well understood; for it has happened, again and again, that a thumb, finger, or toe, thus situated, has been cut off, when, if the nature of the lesion had been properly comprehended, it might have been saved. I would as leave think, in ordinary cases of this kind, of amputating a man's neck for an obstinate ulcer of the lip, chin, or nose, as to remove a finger because it was affected with onychia maligna. It is only when the part has been rendered perfectly useless, as it may be when all its component structures are implicated, that the idea of an operation should be at all entertained.

The treatment of this disease is, I think, on the whole, sufficiently well understood. It consists, as was originally suggested by Mr. Wardrop, in the administration of mercury, to the extent of slight ptyalism, which should be carefully maintained for several successive weeks, or even a full month, if the case is at all obstinate. The best form, according to my observation, is calomel, in the dose of from one to two grains every twelve hours, in combination with a suitable quantity of opium, to prevent it from producing griping and too much action on the bowels. As soon as it is found that there is slight tenderness of the gums, with an increased flow of saliva, and change in the odor of the breath, the mercury should either be omitted, or given in small and less frequent doses. As the effect subsides, the medicine should be again exhibited, and thus it should be continued as long as there seems to be good reason for its employment. Great care should be taken, in all cases, especially when there is a decidedly strumous diathesis, not to carry the remedy to the extent of severe ptyalism. Iodide of mercury and blue mass are also eligible forms of exhibition; but my experience teaches me that calomel is the best.

It need hardly be stated that great attention should always be paid to the bowels and the secretions, both of which are frequently much disordered in this apparently trifling affection. None, however, but the milder purgatives should be employed, such, principally, as are of a vegetable nature. Where the secretions are much deranged, a full dose of calomel, followed in five or six hours by a suitable quantity of castor-oil, cannot fail to be of essential service.

Tonics are indicated where the patient is feeble, and the tendency to reparation tardy and imperfect. Among the best articles of this class are, sulphate of iron and quinine, or iron with some of the vegetable extracts. The diet, in this case, should also be nutritious, but perfectly plain and simple. Where the debility is considerable, and it is evident that it interferes with the reparative process, recourse should be had to wine, wine-whey, brandy, or milk punch.

When there is much pain, preventing sleep, and depriving the patient of appetite, anodynes will be indicated, and should be given in full doses; which, in this, as in most other affections, are far preferable to small and frequently-repeated doses. Where there is dryness of the skin, a good form of exhibition is Dover's powder, or a combination of morphia and antimony.

The local treatment should be of the mildest and most soothing description. When the pain and discoloration are very great, marked benefit may be expected from the application of a few leeches to the end of the finger, or round the root of the nail. Frequent immersion, for fifteen or twenty minutes at a time, of the part in tepid water, slightly impregnated with common salt, has sometimes a very happy effect, apart from its cleansing properties. In case there is much fœtor, a little liquid chlorinate of soda may be added to the water, or a few drops of this fluid may from time to time be sprinkled upon the dressings. As a constant application, I have found nothing to answer as well as an elm-bark poultice, or a linseed cataplasm, with a piece of thin gauze interposed between it and the affected surface, to prevent the poultice from adhering. When the ulcer is very irritable, it may be touched, once a day, with a weak solution of nitrate of silver, or lightly with this article in substance. In all the cases under my charge, I have derived marked benefit from the ointment of the nitrate of mercury, in the proportion of half a drachm to the ounce of opiate cerate. The black and yellow washes have also been serviceable; and various astringent and detergent lotions, as those of zinc, copper, lead, and nitric acid, might, doubtless, be advantageous, though I have nothing to offer in their favor from personal experience. Mercurial fumigations have been highly recommended; I have made repeated use of them in the different stages of my cases, and have received no benefit from them.

It is a good plan to remove the diseased nail as fast as it becomes detached; for its pressure, under these circumstances, not only prevents the contact of the dressings, but is often a source of real suffering. The greatest possible gentleness should be employed in removing it, and also in applying the dressings. It is scarcely necessary to add that the hand should be kept perfectly quiet and in an easy, elevated position.

When the granulations have become healthy, the best applications are the opiate cerate, either alone or in union with a little of the ointment of the nitrate of mercury, and the use of the roller, the pressure of which greatly aids in reducing the swelling, and in restoring the part to its natural form, which, however, it never completely attains, owing, perhaps, to the imperfect reproduction of the nail. Exercise in the open air will materially contribute to the patient's recovery.

